# A Novel In-Line Measurement and Analysis Method of Bubble Growth-Dependent Strain and Deformation Rates during Foaming

**DOI:** 10.3390/polym16020277

**Published:** 2024-01-19

**Authors:** Tobias Schaible, Christian Bonten

**Affiliations:** Institut für Kunststofftechnik, University of Stuttgart, Pfaffenwaldring 32, 70569 Stuttgart, Germany; christian.bonten@ikt.uni-stuttgart.de

**Keywords:** foaming, bubble growth, in-line analysis, elongational deformation, uniaxial and biaxial transient elongational viscosity, strain hardening

## Abstract

Bubble growth processes are highly influenced by the elongational viscosity of the blowing agent-loaded polymer melt. Therefore, the elongational viscosity is an important parameter for the development of new polymers for foaming applications, as well as for the prediction of bubble growth processes. Thus, knowledge of the initial expansion and deformation behavior in dependency on the polymer, the blowing agent concentration, and the process conditions is necessary. This study presents a novel method for the in-line observation and analysis of the initial expansion and deformation behavior within the bead foam extrusion process. For this purpose, nitrogen as the blowing agent was injected into the polymer melt (PS and PLA) during the extrusion process. The in-line observation system consists of a borescope equipped with a camera, which was integrated into the water box of an underwater pelletizer. The camera is controlled by a developed trigger by means of angular step signal analysis of a rotary encoder on the cutter shaft of the underwater pelletizer. Thus, images can be taken at any time during the foaming process depending on the cutter position to the die outlet. It is shown that the developed method provides reliable results and that the differences of the initial expansion and deformation behavior during bubble growth can be analyzed in-line in dependency on real foaming process conditions and the type of polymer used.

## 1. Introduction

Foamed polymers are two-phase systems with a cellular structure consisting of the matrix material (polymer phase) and the blowing agent (gas phase) [1]. Due to the high gas content and the cellular structure, typical properties of polymer foams, such as particularly low density, high insulation capacity (thermal and sound), and high compressibility, are achieved [2,3].

When processing blowing agent-loaded polymer melts, either a chemical (gas generation by chemical reaction) or physical (gas generation by phase transition) blowing agent is used in the extrusion or injection molding process [3]. The physical blowing agent (nitrogen N_2_ was used in this study) is injected into the polymer melt under high pressure, initially forming a two-phase system. Sorption and diffusion processes then dissolve the blowing agent in the polymer melt, resulting in a single-phase solution. The pressure drop in the die (or in the mold) initiates nucleation of the cell nuclei, as the solubility of the blowing agent within the polymer melt is abruptly reduced. Subsequently, the nucleated and growing cell nuclei begin to form bubbles. The formed bubbles continue to grow due to diffusion processes until bubble growth is inhibited by temperature reduction due to cooling processes and thus viscosity increase of the polymer melt [4,5,6,7].

Bubble growth can be described and divided into three main periods according to Taki [8]—viscosity controlled period, transition period, and diffusion controlled period. At the bubble growth beginning, the hydrodynamic forces, and thus the acting pressure difference between the pressure in the gas bubble and the ambient melt pressure, dominate. In this short growth phase, there is hardly any diffusion of the blowing agent from the polymer melt into the gas phase. The growth rate of the bubble in the viscosity-driven period depends to a large extent on the elongational viscosity of the blowing agent-loaded polymer melt, i.e., the flow resistance under elongational deformation and the surface tension at the bubble wall [9]. The higher the elongational viscosity and surface tension, the slower the bubbles grow. The influence of the surface tension is negligible compared to the elongational viscosity of polymer melts [10,11].

In the second, so-called transition period, the pressure in the bubble drops rapidly and approaches the ambient (or melt) pressure. Thereby, the concentration gradient at the phase boundary between the blowing agent-loaded polymer melt and the gas bubble becomes larger and larger, thus enabling the diffusive mass flow of the blowing agent into the gas phase [8].

The third, diffusion controlled period, in which the driving force of the bubble growth is described by the diffusive mass flow of the blowing agent from the polymer melt into the bubble. For all three stages of bubble growth, the driving force in the bubble must be greater than the force at the bubble wall resulting from the elongational viscosity, and thus the resistance to deformation of the blowing agent-loaded polymer melt. The transient elongational viscosity under equibiaxial elongational deformation thus plays an important role in bubble formation, and growth as well as for its prediction [12]. In this context, the elongational viscosity prevailing at the bubble wall during bubble growth and thus the bubble growth behavior depends highly on the structure and type of polymer used, on the time, on the Hencky strain, the strain rate, and the process conditions such as temperature, pressure, and blowing agent concentration [10,13,14]. Therefore, superposition principles can be used to describe the influence of process conditions such as temperature, pressure, and blowing agent concentration on viscosity [15]. 

For the basic description of the transient elongational viscosity at the bubble wall during bubble growth using, e.g., tube models [16,17,18,19], the Hencky strain and the strain rate at the bubble wall during bubble growth are required. If the so-called Trouton ratio is used, the transient as well as the time- and strain rate-dependent deformation behavior during bubble growth is not taken into account, especially for branched polymers [20].

In the literature [8,21,22,23,24,25,26,27,28,29], many investigation methods are known for the analysis of the expansion and bubble growth behavior as a function of the process conditions and the polymer and blowing agent used. However, there are only few, and partly estimated, data in the literature on the elongational deformation behavior that changes at the bubble wall during the bubble growth process and is dependent on time and strain rate. Kropp [30] investigated the strain rate during bubble growth using an extruded foam sheet made of PS and CO_2_. The bubbles formed within the extruded sheet were captured as a function of the haul-off speed using a camera. The strain rate at the bubble wall during bubble growth was estimated to be between 9 s^−1^ and 0.5 s^−1^ according to [30]. However, the strain rate is largely dependent on the cooling of the foamed sheet and the process conditions. Further work estimated the strain rate at the bubble wall during bubble growth to be ≤10 s^−1^ [12] or derived the strain rate at the bubble wall during growth (≤4 s^−1^) from simulations [11,27].

This paper presents an in-line measurement and analysis method within the bead foam extrusion process that allows the characterization of the initial expansion behavior as a function of the process conditions and the polymer used. Thus, for the first time, the overall expansion and the transient elongational deformation behavior caused by overall bubble growth processes can be determined in a real foaming process. In the future, this should allow us to describe the transient elongational viscosity function at the bubble wall by means of, e.g., tube models. By this, the viscosity-dependent bubble growth process may be described holistically using so-called bubble growth models with the deformation data actually prevailing at the bubble wall during bubble growth.

## 2. Materials and Methods

### 2.1. Materials

In this work, the amorphous polystyrene PS 168N from Ineos Styrolution Group GmbH, Frankfurt am Main, Germany, and the semi-crystalline polylactide PLA 2003D IngeoTM biopolymer from NatureWorks LLC, Minnetonka, MN, USA, are used for the investigations. In this context, PS represents a material widely used in various industrial sectors for foaming applications. Whereas PLA is becoming increasingly important as a biobased and biodegradable material for foaming applications, it needs to be modified due to the low viscosity and melt strength. The modification method used was developed at IKT and is described in [31]. After modification, PLA has an increased elongational viscosity and shows strain hardening under deformation. The modification of PLA thus changes the constitution of the polymer chains from a linear (unbranched) to a branched or long-chain branched constitution. This fundamentally changes the flow behavior under shear or elongational deformation [15,19].

### 2.2. Rheological Characterization 

The characterization of the PS and PLA melt under a shear deformation was performed using the Discovery HR-2 rotational rheometer from TA Instruments, New Castle, DE, USA in the plate–plate configuration. The so-called complex viscosity under shear deformation was analyzed by measurements in the frequency sweep mode in the range from 628 rad/s to 0.001 rad/s at 180 °C and 220 °C. The deformation used was set to 5% in the linear viscoelastic region for all measurements. 

The transient uniaxial elongational viscosity is characterized using the Sentmanat Extension Rheometer (SER, [32]) from TA Instruments, New Castle, DE, USA. For this purpose, test specimens (length 18 mm, thickness 0.7 mm, and width 10 mm) were prepared using a compression molding process. To characterize the transient uniaxial elongation viscosity, the so-called extensional stress test is applied. For this purpose, the stress increase is measured as a function of increasing Hencky strain (from 0 to a maximum of 3.8) and thus increasing time under a constant temperature (180 °C and 220 °C) and strain rate (12 s^−1^, 2 s^−1^, 0.05 s^−1^).

### 2.3. In-Line Observation Method within the Underwater Pelletizing Process

**Figure 1:**
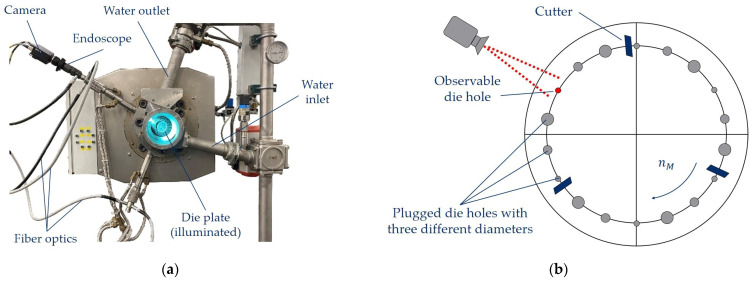
Illustration of the modified pelletizing unit of the underwater pelletizer (**a**) and schematic drawing of the die plate with the observed die hole during processing (**b**).

A single-screw extruder (Ø60 × 30D) with a grooved barrel was used to process the blowing agent-loaded polymer melt. Gaseous nitrogen (N_2_) was mass flow-control injected into the polymer melt using the DSD 500 gas metering system from Linde GmbH, Pullach, Germany. The blowing agent-loaded polymer melt was extruded into the so-called water box through a die plate of the Sphero 70 underwater pelletizing system from Maag Deutschland GmbH, Xanten, Germany (see Figure 1) and immediately cut off by rotating knives. The water box of the underwater pelletizer has been modified to provide direct visual access to the die outlet and thus the holes on the die plate. By this means, the expansion behavior of the blowing agent, and thus the determination of the transient elongational deformation behavior at the bubble wall, was observed. The visual in-line observation system consists of the GS3-U3-23S6M-C camera from Flir, Wilsonville, OR, USA, a TVA-HD-35 lens from Endo Industrial GmbH, Spaichingen, Germany, and a rigid 8 mm endoscope from Karl Storz, Tuttlingen, Germany.

The control and triggering of the camera as well as the data acquisition were performed by means of LabView 2019 from National Instruments, Austin, TX, USA. The triggering of the camera works by using the angular position of the cutter shaft and thus of the knives in relation to the observed die hole on the die plate using the incremental encoder type BHG 1P.05A16384-E2-A from Baumer, Frauenfeld, Switzerland. Due to the resolution and analysis of 65.536 angular pulses per revolution of the cutter, a maximum angular resolution of 0.005° can be achieved with the developed system. This means that an image can be recorded for any angular position of the cutter shaft, and thus of the knives in relation to the observed die hole on the die plate. Thus, the entire pellet formation process and therefore the initial expansion and elongational deformation behavior of the blowing agent-loaded polymer melt can be observed and analyzed in-line at any time during processing.

**Figure 2:**
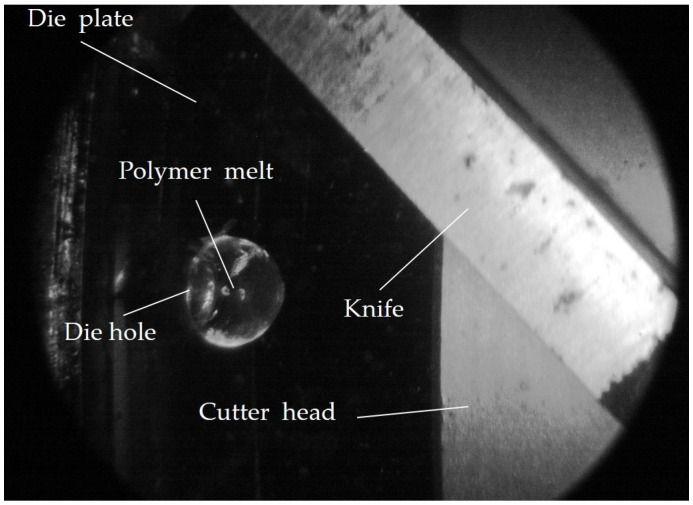
Exemplary image taken with the in-line observation method at a random time step during underwater pelletizing.

The die plate has a total of 18 die holes (see Figure 1b) with three different hole diameters (2.3 mm, 2.7 mm, and 3.1 mm), each of which can be individually closed or opened by pins. One hole with a diameter of 2.3 mm was in-line observed (see Figure 2). It is important to note that, for each complete rotation of the cutter, exactly one image is captured at a defined angular position of the cutter to the in-line observed die hole. After one complete revolution of the cutter, the counter is cleared and thus a new pellet is observed at the same or at another angular position. It is assumed that the pelletizing process is in a stationary state, which means that the pelletizing process and thus the pellet formation is identical for each revolution.

With the in-line observation method, images can thus be taken at any time during pelletizing and therefore during the expansion of the blowing agent in the foaming process. In addition, the initial expansion behavior (directly after nucleation and thus within the initial and viscosity-determined time range of bubble growth) of the blowing agent in the blowing agent-loaded polymer melt is possible without the influence of a back pressure (approximately 1 bar water pressure) and under nearly isothermal conditions inside the pellet during formation and pelletizing [10,33] (main cooling starts after the pellet is cut at the die hole). Furthermore, if the process conditions of the pelletizing process are kept constant, the influence of extrusion conditions (temperature, pressure, and blowing agent concentration) on the initial expansion and elongational deformation behavior at the bubble wall can be analyzed and compared between different polymers. Thus, in particular the first viscosity-driven bubble growth period can be characterized.

### 2.4. Extrusion and Underwater Pelletizing Process Parameters 

Three knives were used for pelletizing at a constant drive shaft speed of 3000 min^−1^. This provides a relatively long observation time while maintaining real pelletizing conditions. The water temperature in the water box is kept constant at 80 °C at a water flow rate of 25 m^3^/h. The die plate temperature is kept constant at 300 °C to avoid local freezing of the blowing agent-loaded polymer melt at the die hole due to the water temperature. 

The screw speed of the extruder was set to 30 min^−1^ and the extrusion temperatures were set to 220 °C and 240 °C for PS and to 200 °C and 220 °C for PLA. Under these process conditions, no local freezing of the die hole was observed under any process condition. At temperatures below 220 °C, local and recurrent die hole freezing was observed for PS. A higher temperature than 220 °C is not applicable for PLA due to thermal degradation. The selected die plate temperature of 300 °C is an exception, since the residence time of the melt in the die plate is approximately 5 ms (calculated according to [34]). Such a short time is negligible in terms of thermal degradation of PLA at 220 °C and also helps in preventing local freezing of the die hole. Due to the constant screw speed, it is also ensured that both polymers used are subjected to a constant shear stress during extrusion. This allows direct comparison of each polymer as a function of the process parameter variation, as well as between PS and PLA at 220 °C.

The dosing mass flow of the injected nitrogen had to be adjusted between the experiments with PS and PLA in order to achieve comparable N_2_ concentrations because of the constant screw speed and the resulting mass flow rates (see Table 1). 

Due to the control range and the control accuracy of the dosing station, a maximum deviation of 0.01 wt.-% N_2_ between the experiments with PS and PLA at a comparable blowing agent concentration was achieved. This allows a direct comparison of the expansion and deformation behavior at an almost constant blowing agent concentration between the experiments with PS and PLA. 

### 2.5. Analysis of the Initial Expansion and Deformation Behavior at the Bubble Wall

**Figure 3:**
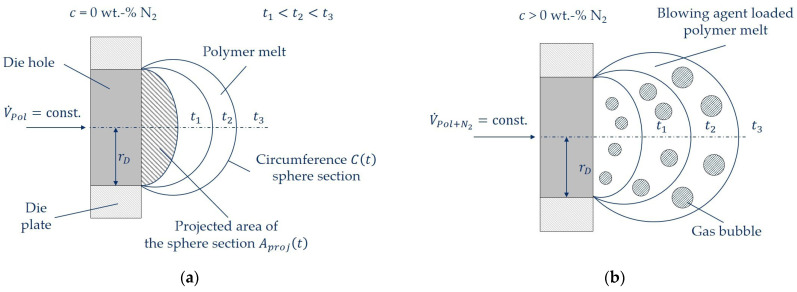
Schematic representation of the model for the analysis of the initial expansion and deformation behavior at exemplary times without a blowing agent concentration as reference (**a**) and for blowing agent-loaded polymer melts with otherwise unchanged process conditions (**b**).

Figure 3 shows schematically the in-line observed die hole on the die plate for an experiment without (a) and with a blowing agent (b) at three exemplary times between two successive cuts. In Figure 3b it can be seen that the four exemplarily shown bubbles at each time from $t_{1}$ to $t_{3}$ grow with increasing time due to bubble growth processes. Thus leading to an increasing expansion of the blowing agent in the overall observed volume $V_{Pol+N_{2}}\left(t\right)$ of the blowing agent-loaded polymer melt at each time observed in-line. It is important to note that single bubbles cannot be observed in-line. At all times, the overall volume of the polymer melt $V_{Pol}\left(t\right)$ or the blowing agent-loaded polymer $V_{Pol+N_{2}}\left(t\right)$ is observed in-line, depending on if a blowing agent is used or not under unchanged process conditions (such as shown in Figure 3). Therefore, it is important to point out that it is not the aim of this work to achieve the lowest possible foam density or a perfectly homogeneous foam structure, but to characterize and holistically describe the overall expansion and deformation behavior (Hencky strain and strain rate) of all growing bubbles in the polymer melt within the initial and thus viscosity-driven bubble growth process for the first time in a real foaming process.

The observation of the pellet formation in underwater pelletizing without a blowing agent (see Figure 3a) was fundamentally developed by Geiger and Grünschloss [33,35] and Kast et al. [36,37] at the Institut für Kunststofftechnik (IKT) at the University of Stuttgart. These basic principles were taken up here, extended, and applied for the first time to a blowing agent-loaded polymer melt (see Figure 3b) on the basis of the model concept described in the following.

The time available for recording and describing the deformation behavior at the bubble wall during bubble growth is that between two successive cuts $t_{S}$ of the knives. This has been calculated as a function of the drive shaft speed $n_{M}$ and the number of knives used $a_{M}$ according to Equation (1).

(1) $$t_{S}=\frac{1}{a_{M}\cdot n_{M}}$$

The observation of pellet formation and expansion of N_2_ at the die hole is carried out in the time range $0\leq t\leq t_{S}$. Due to the very short cutting times, the pellet formation process can be considered as almost isothermal in the time range $0\leq t\leq t_{S}$ and at a backpressure of approx. 1 bar [10,33]. 

In the time range $0\leq t\leq t_{S},$ the projected area $A_{proj}\left(t\right)$ (see Figure 3) of the volume of the polymer melt $V_{Pol}\left(t\right)$ emerging from the die hole into the water box and the volume of the blowing agent-loaded polymer melt $V_{Pol+N_{2}}\left(t\right)$ is visually in-line observed and analyzed. Assuming that the exiting volume is spherical (and thus assuming equibiaxial elongational flow), a radius and thus a spherical volume at each time can be calculated from the projected area. The fraction of the volume of the expanded N_2_ $V_{N_{2}}\left(t\right)$ at each time in the time range was calculated according to Equation (2) as the difference between an experiment without blowing agent and an experiment with blowing agent under otherwise constant process conditions. The evaluation of the projected area $A_{proj}\left(t\right)$ is performed with the image processing and image analysis software Fiji ImageJ, Version 2.9.0.

(2) $$V_{Pol+N_{2}}\left(t\right)=V_{Pol}\left(t\right)+V_{N_{2}}\left(t\right)$$

In addition, a change in the specific volume can be neglected due to the blowing agent concentrations used (c ≤ 0.34 wt.-% N_2_). Furthermore, the upstream pressure of the die plate is reduced due to the decreasing viscosity with an increasing blowing agent concentration [15]. Nevertheless, it can be assumed that the mass throughput remains constant due to the grooved barrel extruder used [2]. 

The deformation in Figure 3 (polymer melt and N_2_ expansion in the melt) is analyzed in the time range $0\leq t\leq t_{S}$ and as a function of the process conditions and the polymer used. The proportions are summed in the case of superimposed Hencky strain [38]. An equibiaxial elongational flow during pellet formation and bubble growth is assumed. 

To describe the total Hencky strain of the blowing agent-loaded polymer melt $\varepsilon_{Pol+N_{2}}\left(t\right)$ according to Equation (3), the proportion of the Hencky strain of the polymer melt $\varepsilon_{Pol}\left(t\right)$ and of the expanding blowing agent $\varepsilon_{N_{2}}\left(t\right)$ is required for each observed time.

(3) $$\varepsilon_{Pol+N_{2}}\left(t\right)=\varepsilon_{Pol}\left(t\right)+\varepsilon_{N_{2}}\left(t\right)$$

The corresponding proportions in Equation (3) are calculated from experiments with and without a blowing agent concentration under otherwise constant process conditions according to Equation (4) in the time range $0\leq t\leq t_{S}$. For this purpose, the corresponding proportions from Equation (3) are calculated as the logarithmized ratio of the change in length $l\left(t\right)$. Therefore, the projected time-dependent circumference of the sphere section $C\left(t\right)$ of the polymer melt or of the blowing agent-loaded polymer melt emerging from the die hole (see Figure 3) is analyzed and set in relation to the length $l_{0}$ at time t = 0 s. This corresponds to twice the die hole radius $r_{D}$ since, at time t = 0 s, no melt has yet emerged from the die hole into the water box. The correlating strain rate of the respective components from Equation (3) at each time t in the time range $0\leq t\leq t_{S}$ are described accordingly with Equation (5).

(4) $$\varepsilon \left(t\right)=ln\left(\frac{l\left(t\right)}{l_{0}}\right)=ln\left(\frac{C\left(t\right)}{2\cdot r_{D}}\right)$$

(5) $$\dot{\varepsilon }\left(t\right)=\frac{\varepsilon \left(t\right)}{t}$$

Thus, the Hencky strain and the strain rate of the emerging polymer melt or the blowing agent-loaded polymer melt can be characterized and analyzed as a function of time, process conditions, and the polymer used. In addition, the deformation due to the expansion of the blowing agent can be described, thus representing the average prevailing Hencky strain at the bubble wall $\varepsilon_{N_{2}}\left(t\right)$ during bubble growth. 

After the pelletizing process, the projected area $A_{proj}\left(t\right)$ of the unfoamed and foamed pellets is examined. For this purpose, 30 pellets per process condition are scanned as an 8-bit grayscale image. The evaluation of the projected pellet area is also performed with the software Fiji ImageJ, Version 2.9.0. If the projected pellet area is additionally converted into a circular area, and thus the spherical circumference is analyzed, the Hencky strain and the strain rate can be calculated according to Equations (4) and (5) at 4520 ms. This corresponds to the removal of the pellets from the separator of the underwater pelletizing process.

The main advantage is that the reference of the model for the determination of the deformation at t = 0 s is for the first time exactly defined by the die hole radius (Equation (4)). In contrast, this is not the case in the literature. Thus, the starting point of the observation of the expansion in, e.g., Kropp [30] depends on the earliest observable time of several expanding bubbles in an extruded sheet and the prevailing process conditions. In, e.g., Ramesh and Lee [11], a simulated initial radius is used to calculate the elongation deformation, which, however, has not been determined experimentally and thus cannot be proven. In addition, the influence of the strand expansion is automatically taken into account in this work at each time of each experiment with and without blowing agent, because of the analysis of the circumference $C\left(t\right)$ of the sphere section. This is important because strand expansion has an influence on the expansion and thus the deformation behavior [39].

## 3. Results and Discussion 

### 3.1. Transient Uniaxial Elongational Viscosity of PS and PLA 

**Figure 4:**
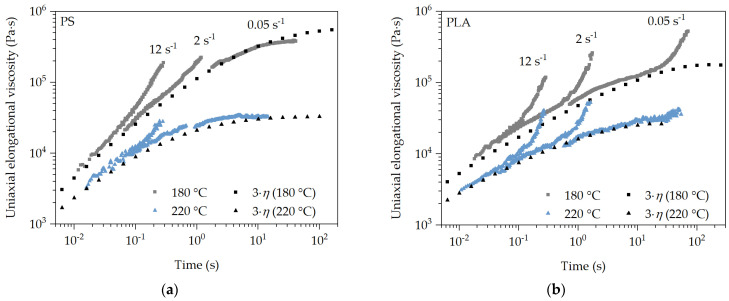
Analysis of the transient uniaxial elongational viscosity profile of PS (**a**) and PLA (**b**) at exemplary SER measurement conditions in relation to the uniaxial Trouton ratio (3·η).

The characterization of the flow behavior of the polymer melt under elongational deformation is very important for the analysis of the expansion behavior of the blowing agent in the polymer melt and thus the deformation behavior at the bubble wall during bubble growth. Especially since these are transient processes and depend mainly on the process conditions during foaming. The measured transient elongational viscosity of PS (a) and PLA (b) at different temperatures, strain rates, and over a wide Hencky strain range is shown in Figure 4 as the average of three measurements at each setting. It is important to note that each measurement is performed at a constant temperature and strain rate with an increasing Hencky strain. In addition, the uniaxial Trouton ratio from shear rheological measurements is shown. The typical temperature and strain rate dependency for polymer melts is presented for all measurements. It can be seen that strain hardening occurs at strain rates of 12 s^−1^ and 2 s^−1^ for PS and PLA. The effect of strain hardening decreases with increasing temperature and decreasing strain rate. However, it can be seen that this effect is absent at both temperatures and there is good agreement over time with the uniaxial Trouton ratio (3 η) for PS at the strain rate of 0.05 s^−1^. This flow behavior is equally evident for PS at 2 s^−1^ and 220 °C. Therefore, it can be assumed that there is no strain hardening caused by branched polymer chains in PS, and only a stress increase at lower temperatures and higher strain rates. This can be attributed to the reduced free volume and thus inhibited chain mobility at decreasing temperatures and a very rapidly increasing deformation at high strain rates. In this case, the polymer chains do not have sufficient time to disentangle and slide off each other at an increasing Hencky strain over time, which is why the stress increases exponentially. Since this deformation behavior does not occur at low strain rates regardless of the temperature, it can be assumed that PS has linear polymer chains. This assumption is supported by the literature [40,41].

With PLA, on the other hand, strain hardening can be observed at all temperatures and strain rates, which can be attributed to the modification of PLA. Strain hardening is less pronounced with a decreasing strain rate and an increasing temperature, since the time and chain mobility increases and thus untangling and sliding off each other of the polymer chains is easier with an increasing deformation. Furthermore, the uniaxial Trouton ratio (3 η) does not agree with the transient uniaxial elongational viscosity measurements from the time of occurrence of strain hardening. This can be explained by the branched polymer chains of the modified PLA. In this case, the branched polymer chains cannot disentangle and slide off each other fast enough under deformation, which is why the stress increases exponentially with increasing Hencky strain at all strain rates.

### 3.2. In-Line Observation Method and Evaluation of Its Accuracy and Reproducibility

**Figure 5:**
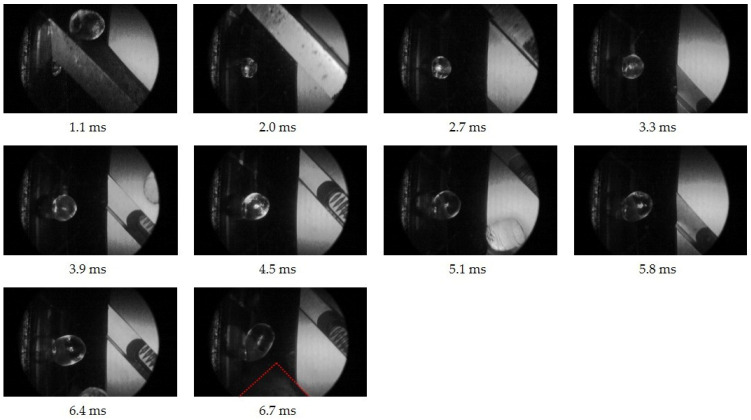
In-line observation at ten exemplary times between two cuts in the underwater pelletizing process of PS at 220 °C and 0 wt.-% N_2_ to analyze the expansion and thus the deformation behavior.

Based on the selected process conditions in the underwater pelletizing process (see Section 2.4), the visual in-line observation and analysis of the expansion and deformation behavior is given for all experiments at all times between two cuts. This is exemplarily shown in Figure 5 for PS in the time range $0\leq t\leq t_{S}$. 

At 1.1 ms after the polymer melt exits the die hole, the knife releases the die hole with the old, cut-off pellet still in contact with the knife. At 6.7 ms, the polymer melt is just before been cut and deformed in the water due to the approaching knife (marked in red). However, in the time range observed in-line, the pellet formation behavior can be characterized. In the case of blowing agent-loaded polymer melts, the average expansion behavior of the blowing agent and thus the average deformation behavior at the bubble wall can be analyzed in-line as well. Therefore, the reference experiment with no blowing agent is performed with otherwise unchanged process parameters, as described above. 

**Figure 6:**
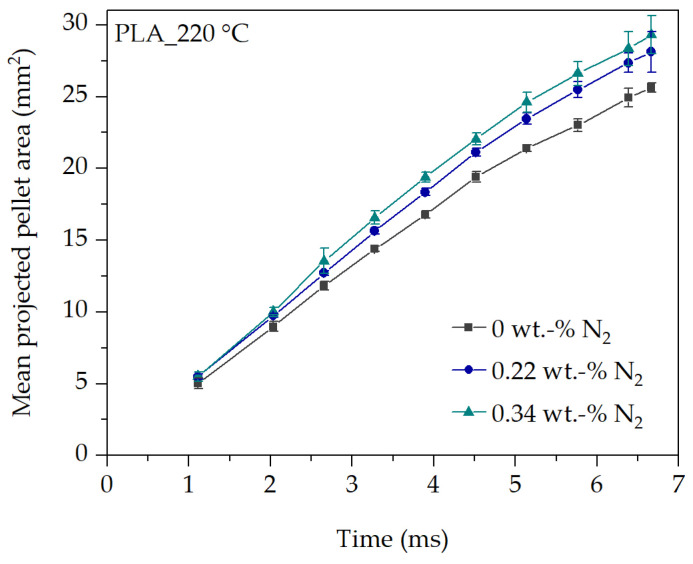
Analysis of the mean projected pellet area in dependency of N_2_ concentration for PLA at 220 °C.

The accuracy and reproducibility of the in-line observation method is given by the fact that 30 images are recorded per process parameter changed and at each observation time (from 1.1 ms to 6.7 ms). In addition, 30 exemplary foamed pellets are analyzed after pelletizing (at time 4.52 s after the cut). Therefore, the evaluation of the projected pellet area represents an average value of 30 individually analyzed pellets at each observation time and process parameter setting. This is shown in Figure 6 for PLA at 220 °C and for different N_2_ concentrations with otherwise constant process conditions.

It can be seen that the mean projected pellet area increases over time (900 evaluated images in Figure 6). This seems plausible for physical reasons. The observed projected pellet area and thus the volume at the die hole exit must increase with time due to the continuous mass throughput of the extruder. Furthermore, an increasing expansion and thus an increased projected pellet area is expected at any time with an increasing blowing agent concentration, compared to a lower one. 

Due to the fact that each captured image corresponds to a new pellet in the cutting process at each observed time, the reproducibility of the test sequence and the in-line observation method can be evaluated as well.

**Figure 7:**
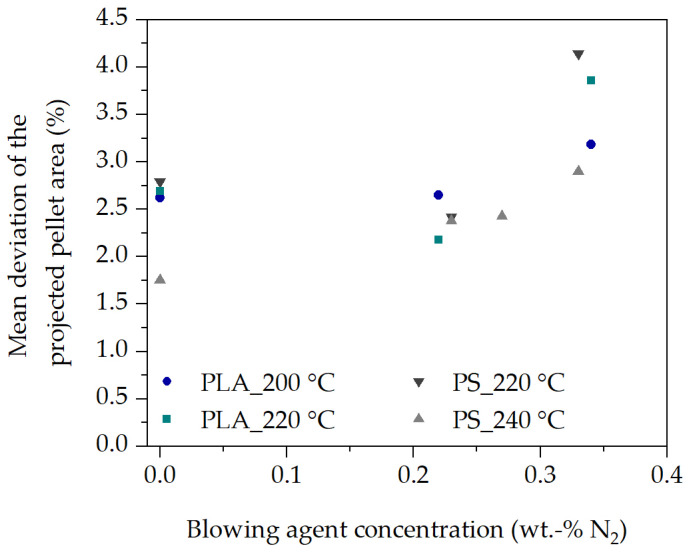
Investigation of the reproducibility of the in-line observation method for PLA and PS.

In Figure 7, each data point corresponds to the mean deviation of each experiment with ten observation times each, as shown in Figure 6. The reproducibility is 2.8 % averaged over all experiments and observation times. For this purpose, a total of 3900 images and thus independent pellets were analyzed during pellet formation and expansion of N_2_. This clearly demonstrates the reproducibility of the experimental procedure, the in-line observation method, and the repeatable evaluation of the projected pellet area during pellet formation and blowing agent expansion in the polymer melt.

### 3.3. Analysis of the Initial Expansion Behavior of the Blowing Agent during Foaming 

**Figure 8:**
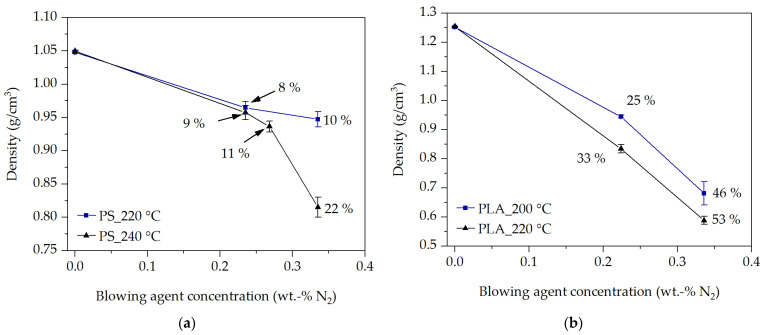
Pellet density at the end of the underwater pelletizing process at 4.52 s for PS (**a**) and PLA (**b**) at two temperatures.

In the simplest case, the resulting expansion of the blowing agent in the blowing agent-loaded polymer melt can be analyzed from the density of the pellets at the end of the pellet foaming process (after 4520 ms) as a function of the process conditions. The density of the pellets in Figure 8 was determined using the hydrostatic buoyancy method.

The expansion behavior of the blowing agent, and thus the resulting pellet density, can be explained on the basis of viscosity. With an increased temperature and blowing agent concentration the viscosity at the bubble wall is reduced [15], whereby bubbles can grow more easily and quickly due to the lower resistance in the surrounding blowing agent-loaded polymer melt. This was also shown in [27] for other PS and in [31] for other PLA types, as well as in [42] for polymers with low melting temperature and low viscosity. In addition, with an increased N_2_ concentration, more blowing agent is available. This further reduces density. On the other hand, as the temperature decreases and thus the viscosity increases, the pressure drop increases as the polymer melt flows through the die plate at a constant extrusion volume rate. This should increase the nucleation rate [6,8], leading to an increasing density reduction as more bubbles nucleate, as shown by Stange [5] or Wang [12]. However, in Figure 8, an increasing density reduction with increasing temperature at the same N_2_ concentration is observed in all experiments compared to a decreasing temperature and thus increasing pressure drop. 

In contrast, the behavior observed in Figure 8 was also shown by Lee et al. [24] in foam extrusion. Therefore, it can be assumed that the pressure drop is decisive for the nucleation but not for the bubble growth and thus the expansion behavior. This is also supported by the relatively small pressure differences (extrusion pressure) within the experiments with PS and PLA between the selected extrusion temperatures and N_2_ concentrations (PS: between 96 bar and 120 bar; PLA: between 146 bar and 176 bar). Therefore, temperature, blowing agent concentration, and thus the equibiaxial elongational viscosity at the bubble wall play important roles in bubble expansion and growth. 

**Figure 9:**
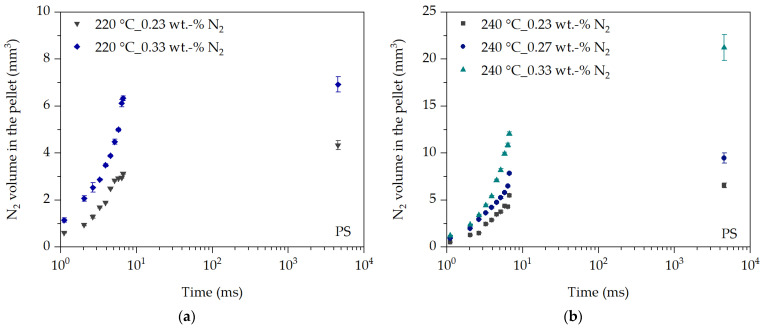
Initial expansion behavior over time of N_2_ in PS at 220 °C (**a**) and 240 °C (**b**).

This is particularly evident in the analysis of the initial expansion behavior of the evaluated nitrogen volume $V_{N_{2}}\left(t\right)$ over time in Figure 9. The initial expansion behavior of N_2_ in PS follows the relationship of the resulting density in Figure 8 as a function of temperature and N_2_ concentration, and thus the prevailing viscosity at the bubble wall. This is clearly seen during the nearly isothermal cutting process in the time range between 1.1 ms and 6.7 ms. At the same time, the expansion behavior at the same N_2_ concentration and at 240 °C is clearly increased at all times compared to 220 °C. This behavior is also evident between the last observation time of the cutting process (6.7 ms) and the removal of the pellet from the process after about 4520 ms. For example, at 220 °C and 0.33 wt.-% N_2_, the gas volume of the blowing agent increases by only 9%, whereas, at 240 °C and the same N_2_ concentration, the gas volume increases by approx. 57% due to the reduced viscosity at the bubble wall at elevated temperatures. Thus, increasing viscosity inhibits the expansion of the blowing agent. This effect is enhanced by the cooling of the pellets in the time range between 6.7 ms and 4520 ms. 

The effect of the N_2_ concentration on the expansion behavior is also shown in Figure 9. The higher the N_2_ concentration, the greater the expansion at each observation time at the same temperature. This behavior is also shown, for example, by Wang [12] or Lee et al. [24]. The influence of the pressure drop, and thus the influence of the nucleation rate, can almost be neglected, since the pressure in the blowing agent-loaded experiments with PS at 220 °C is between 110 bar and 112 bar and at 240 °C between 96 bar and 99 bar. Based on this, the sensitivity of the in-line observation method can be evaluated with the additional N_2_ concentration of 0.27 wt.-% N_2_ at 240 °C. It can be seen that the expansion behavior at 0.27 wt.-% N_2_ is between that at 0.23 wt.-% N_2_ and 0.33 wt.-% N_2_, which was expected. This is further confirmed by a comparison based on the ratio of the resulting density at 240 °C as a function of the N_2_ concentration in Figure 8. 

**Figure 10:**
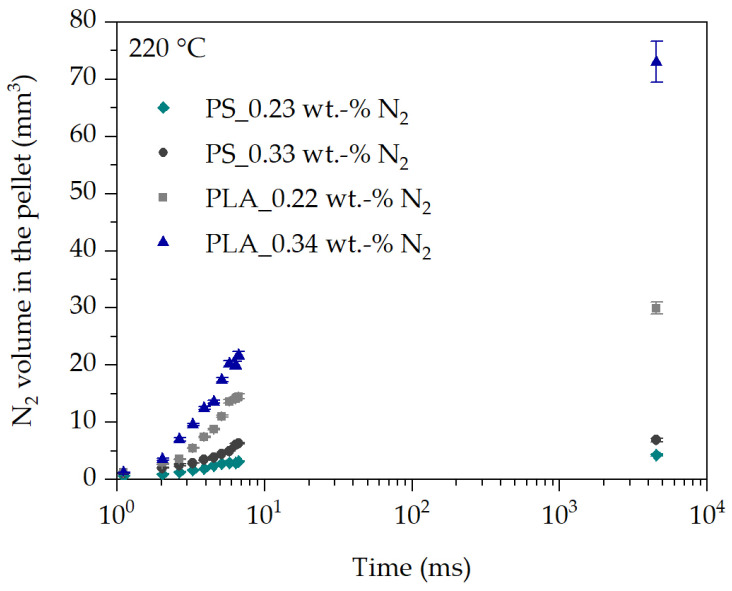
Comparison of the initial expansion behavior over time of N_2_ in PLA and PS at 220 °C.

The relationships discussed based on the expansion behavior of N_2_ in PS also apply to the analysis of the expansion behavior of N_2_ in PLA at 220 °C and the two blowing agent concentrations of 0.22 wt.-% N_2_ and 0.34 wt.-% N_2_ in Figure 10. However, the expansion behavior of N_2_ in PLA is significantly increased at each time compared to PS.

**Figure 11:**
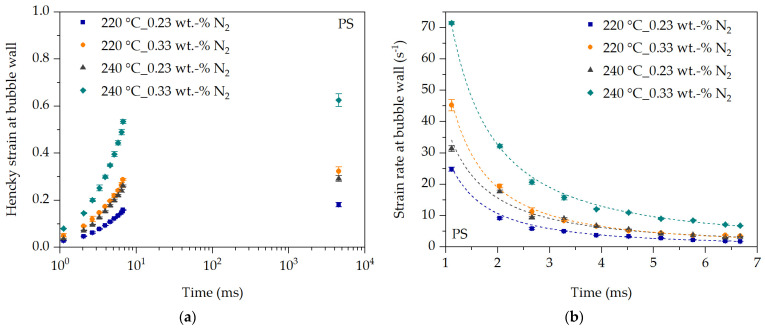
Hencky strain (**a**) and strain rate (**b**) over time during expansion of the blowing agent at the bubble wall for PS in dependency of temperature and blowing agent concentration.

**Figure 12:**
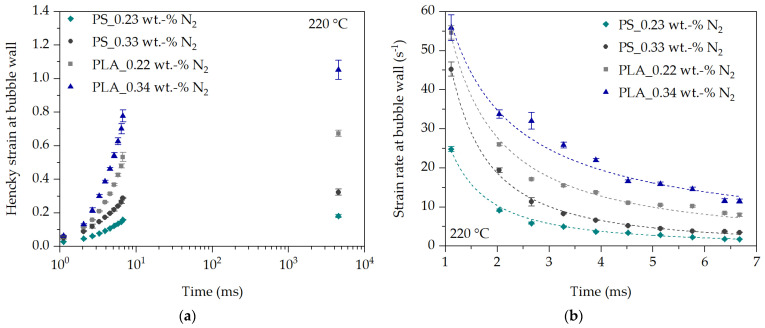
Hencky strain (**a**) and strain rate (**b**) over time during expansion of the blowing agent at the bubble wall for PS and PLA in dependency of temperature and N_2_ concentration.

One reason for the increased expansion behavior of N_2_ in PLA could be the increased extrusion pressure of about 36 bar (32% higher) compared to PS at both N_2_ concentrations and otherwise identical process conditions during underwater pelletizing process in Figure 10. This would require more bubbles to nucleate and subsequently grow in PLA. Furthermore, it can be clearly seen in Figure 4b that the modification of PLA results in branched polymer chains. The branching of the polymer chains causes strain hardening at elongational deformation. This is accompanied by an increase in melt strength of PLA, which can prevent bubble collapse, gas bubble rupture and thus gas loss, and bubble coalescence. With strain hardening, the elongational viscosity of PLA should increase and thus should hinder bubble growth as well. On the other side, strain hardening begins at elevated Hencky strain. Thus, at the beginning of bubble growth no strain hardening should occur since the Hencky strain increases from zero and the strain rate decreases over time of bubble growth, as shown in Figure 11 and Figure 12. 

Lastly, it is shown that PLA has a lower zero shear viscosity at 220 °C compared to PS (PLA: 26,337 Pa∙s and PS: 33,715 Pa∙s). This should lead as well to an increased expansion behavior of N_2_ in PLA compared to PS at the same temperature and blowing agent concentration. A comparison of the transient elongational viscosity of PLA and PS cannot be made to describe the behavior in Figure 10, since the transient elongational viscosity during bubble growth is highly dependent on the changing Hencky strain and strain rate over time.

Based on this, it is reasonable to assume that the expansion behavior at the same temperature and N_2_ concentration is dependent on the constitution of the polymer chains and the associated flow behavior under elongational deformation. This is also supported by the literature [5,12]. This clearly shows the importance of knowledge about the deformation behavior during bubble growth processes in order to be able to describe and predict the transient equibiaxial viscosity function during bubble growth in the future. 

### 3.4. Deformation Analysis at the Bubble Wall during Bubble Growth 

The initial deformation behavior at the bubble wall during bubble growth can be characterized with the initial expansion behavior of the blowing agent in the blowing agent-loaded polymer melt. For this purpose, the prevailing Hencky strain and the strain rate at the bubble wall during the expansion of the blowing agent are determined using the definition described in Section 2.5. At time t = 0 ms, no polymer melt has yet emerged from the die hole into the water box leading $l\left(t\right)$ = $l_{0}$ and thus the Hencky strain and strain rate are considered to be zero. The strain rate abruptly increases after t > 0 ms and then decreases with increasing Hencky strain (see Figure 11 and Figure 12). 

The Hencky strain at the bubble wall increases with time for PS, as shown in Figure 11a. This seems logical since the expansion behavior increases with time. Thus, the Hencky strain at the bubble wall must increase with time due to the expansion of the blowing agent. Accordingly, the Hencky strain at the bubble wall follows the temperature and N_2_ concentration dependencies discussed earlier. The same applies to the strain rate at the bubble wall calculated from the Hencky strain and time.

The strain rate (approximated by a growth function) at the bubble wall during expansion of N_2_ is shown in Figure 11b. The strain rate drops almost abruptly within the first 4 ms. If the last observation point after 4520 ms is included, the strain rate at the bubble wall drops to almost zero. One reason is that the blowing agent expands abruptly after exiting the die hole, thus causing rapid bubble growth within the first milliseconds. As the bubbles continue to grow, more and more blowing agent is required from the surrounding blowing agent-loaded polymer melt to maintain the bubble growth process. However, the bubble growth process is hindered by the increasing equibiaxial elongational viscosity at the bubble wall, which increases with increasing Hencky strain and decreasing strain rate during the time of bubble growth (see also Figure 4). As a result, bubble growth is increasingly inhibited, which means that the strain rate at the bubble wall must decrease with time despite a continuous increase in Hencky strain at the bubble wall and thus bubble expansion.

The comparison of the deformation behavior at the bubble wall for PS and PLA at 220 °C is shown in Figure 12 and clearly shows that the initial Hencky strain and strain rate at the bubble wall is greater in PLA than in PS at each observation time. This can be explained in a similar way to the expansion behavior described in Figure 10.

A direct comparison of the initial and in-line analyzed deformation behavior at the bubble wall with literature data is not possible, since there are no studies in the literature for the initial time period and thus immediately after nucleation observed deformation behavior in the real process. In addition, as shown above, the expansion rate and thus the strain rate depend on the polymer used (flow behavior) and the process conditions (temperature, pressure, blowing agent concentration). However, if the time range between 6.7 ms and 4520 ms is considered, at least an estimate of the plausibility of the determined strain rates can be made with literature data. For example, Kropp [30] states the strain rate at the bubble wall between 9 s^−1^ and 0.5 s^−1^ in an extruded and foamed PS sheet after die exit over time. Tuladhar and Mackley [27] simulated the strain rate at the bubble wall (pentane in PS) between 4 s^−1^ and 0.1 s^−1^ in the time range between 50 ms and 10 s, and Ramesh and Lee [11] (n-butane in PE-LD) state the strain rate between 2.5 s^−1^ and 0 s^−1^ in the time range between 500 ms and 4 s after nucleation. A comparison with the determined strain rates in Figure 12b shows that they are in a similar strain rate range in the time range of 6.7 ms and 4,520 ms. For example, the strain rate shown in Figure 12b drops from a maximum of 11.5 s^−1^ (PLA_220 °C_0.34 wt.-% N_2_) and a minimum of 1.8 s^−1^ (PS_220 °C_0.23 wt.-% N_2_) at the observation time 6.7 ms to about 0.06 s^−1^ to 0.005 s^−1^ at the observation time 4520 ms. 

## 4. Conclusions

In this work, a novel experimental in-line observation method was demonstrated under real foam extrusion process conditions to characterize the initial and thus viscosity-driven expansion behavior of the blowing agent and therefore the bubble growth behavior. The method has been successfully demonstrated to have very good repeatability and sensitivity for analyzing the initial expansion, and ultimately the elongational deformation behavior, as a function of the extrusion process conditions and the type of polymer used. It has been shown experimentally for the first time that the Hencky strain and the strain rate at the bubble wall during initial bubble growth exhibit a highly transient and process condition-dependent behavior. This directly confirms the assumptions in the literature that the bubble growth process is highly viscosity driven. The results of the analyzed expansion behavior further confirm this. Finally, it was successfully shown that the Hencky strain and the strain rate at the bubble wall during initial bubble growth are significantly higher compared to previous data in the literature, making the dependence on the so-called equibiaxial elongational viscosity at the bubble wall during bubble growth clear. The Hencky strain over time of bubble growth increases with increasing temperature and blowing agent concentration. The strain rate at the bubble wall, on the other hand, drops almost abruptly from very high strain rates within a few milliseconds as the blowing agent-loaded polymer melt exits the die hole.

For the investigation and realistic description of the initial bubble growth behavior, the knowledge of the elongational deformation behavior as a function of the Hencky strain and strain rate at the bubble wall is therefore indispensable for the description of the elongational viscosity profile actually prevailing at the bubble wall during bubble growth. The transient deformation data during bubble growth presented in this work for the first time allow us to determine the transient elongational viscosity function actually prevailing at the bubble wall, e.g., using so-called tube models. Thus, the viscosity-driven bubble growth behavior in the real foam extrusion process could be realistically predicted.

## Figures and Tables

**Table 1:** Overview of the resulting mass fraction of blowing agent in regards of the mass flow rate of the polymer at 30 min^−1^ and injection mass flow rate of N_2_ for PS and PLA.

	Polymer Mass Flow Rate (kg/h)	N_2_ Mass Flow Rate (kg/h)	Mass Fraction (wt.-% N_2_)
PS	29.8	0.0; 0.07 and 0.1	0.0; 0.23 and 0.33
PLA	35.7	0.0; 0.08 and 0.12	0.0; 0.22 and 0.34

## Data Availability

Data are contained within the article.
